# Synthesis of Heterobifunctional Protein Fusions Using Copper-Free Click Chemistry and the Aldehyde Tag**

**DOI:** 10.1002/anie.201108130

**Published:** 2012-03-12

**Authors:** Jason E Hudak, Robyn M Barfield, Gregory W de Hart, Patricia Grob, Eva Nogales, Carolyn R Bertozzi, David Rabuka

**Affiliations:** * Departments of Chemistry and Molecular and Cell Biology, Howard Hughes Medical Institute, University of CaliforniaBerkeley, CA 94720 (USA); Redwood Bioscience Inc5703 Hollis St., Emeryville, CA 94608 (USA); Department of Molecular and Cell Biology, Howard Hughes Medical Institute, Lawrence Berkeley National Laboratory, University of CaliforniaBerkeley, CA 94720 (USA)

**Keywords:** antibodies, bioorganic chemistry, click chemistry, oximes, proteins

Heterobifunctional protein fusions are gaining interest as next-generation biopharmaceuticals.^1^–^5^ Combining proteins with disparate functions can enable multidrug therapy with a single chemical entity,^6^, ^7^ add a targeting element to an otherwise nonspecific therapeutic,^8^, ^9^ or improve the pharmacokinetic profile of a rapidly cleared molecule.^10^, ^11^ Indeed, heterobifunctional proteins, such as immunoglobulin G (IgG) Fc domain fusions, are among the top-selling biotherapeutics on the market today.^12^ These biomolecules are primarily generated as genetic fusions. The DNA sequences that encode the individual protein components are fused in tandem to direct the expression of a single polypeptide that comprises the two proteins joined together at their N and C termini, respectively. However, this limited topology is not ideal for every protein combination, as some polypeptides require unmodified termini for optimal bioactivity^13^ or can suffer from expression difficulties as a result of folding and processing issues.^3^, ^14^, ^15^

An alternative approach to generating protein–protein fusions is through chemical conjugation. Native chemical ligation of C-terminal thioesters with β-amino thiols is a powerful method for generating protein–protein fusions,^16^–^18^ but at least one coupling partner must be linked at its terminus. In principle, greater topological diversity can be achieved by introducing bioorthogonal functional groups at specific amino acid side chains of the two proteins.^19^, ^20^ As a recent example, Hutchins et al. expressed a Fab fragment bearing an unnatural keto amino acid to which a maleimide-funtionalized linker was conjugated by oxime formation.^21^ This, in turn, enabled further conjugation to a single cysteine residue that was engineered into a protein toxin. Implicit in this work is the need for a protein–protein coupling reaction with intrinsically fast kinetics, of which thiol to maleimide addition is a paragon example. In this direction, Bundy and Swartz have implemented cell-free protein synthesis to install azide and alkyne amino acids into green fluorescent protein for Cu-catalyzed dimerization.^22^ This approach, however, suffered from low protein expression as well as Cu-induced protein damage.

The strain-promoted 1,3-dipolar cycloaddition of cyclooctynes and azides, also termed the Cu-free azide–alkyne cycloaddition, is a bioorthogonal reaction that is well suited for protein–protein conjugation.^23^–^26^ The cyclooctyne reagent can be tuned for fast kinetics and the reaction proceeds selectively under a wide range of conditions.^27^–^31^ However, harnessing these qualities for heterobifunctional protein conjugate synthesis first requires a practical route for the site-specific introduction of the necessary reactive partners.

The genetically encoded aldehyde tag offers a simple means of site-specific protein functionalization.^32^–^34^ The tag consists of a succinct five-residue sequence (CxPxR) that is recognized by the formylglycine-generating enzyme (FGE). FGE oxidizes the genetically-encoded cysteine residue to the aldehyde-bearing residue formylglycine (fGly) during protein expression in either *E. coli* or mammalian cells (Scheme 1 A).^35^ The aldehyde can then be modified by hydrazone or oxime formation (Scheme 1 B).^36^ Thus, the aldehyde tag serves as a means for site-specific introduction of azides or cyclooctynes onto recombinant proteins through small-molecule linkers. Once installed, these rapidly reacting functional groups enable the assembly of protein–protein conjugates (Figure 1 A). Herein, we employed this approach in the synthesis of heterobifunctional chemical protein fusions of unprecedented complexity; a full length human IgG (155 kDa) coupled to either human growth hormone (hGH, 26 kD) or the maltose-binding protein (MBP, 42 kDa). This site-selective coupling highlights the potential of Cu-free click chemistry in state-of-the-art controlled protein assembly.

**Figure 1:**
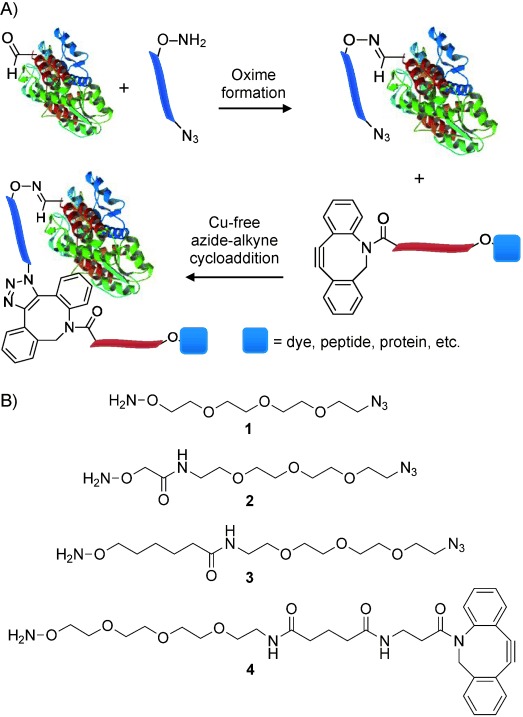
Bifunctional “click” linkers. A) Aldehyde-tagged, fGly-containing proteins are first treated with an azido-aminooxy bifunctional linker. Cu-free click chemistry can then be performed for covalent attachment to any DIBAC-functionalized molecule. B) Heterobifunctional linkers for introducing azides and cyclooctynes onto aldehyde-tagged proteins.

**Scheme 1:**
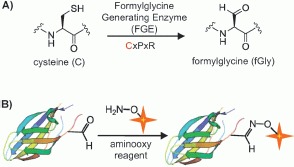
Aldehyde tag enables site-specific protein modification. A) FGE recognizes the sequence CxPxR and converts Cys into fGly by oxidation of the sulfhydryl group to an aldehyde. B) The aldehyde reacts with an aminooxy reagent to form a stable oxime.

To expand on previous reports of fGly conjugation, we initially identified the optimal conditions for oxime formation on aldehyde-tagged recombinant proteins. MBP was chosen as a model monomeric globular protein, whereas human IgG1 (hIgG) served as a more complex and clinically relevant conjugation substrate. Additionally, both MBP and hIgG demonstrate more than 90 % Cys-to-fGly conversion when expressed in bacterial^32^ and mammalian^33^ cell hosts, respectively (see the Supporting Information for conversion analytical data). Conjugations with aminooxy Alexa Fluor 488 (AO-AF488) in various buffers were strongly pH dependent, with yields reaching over 70 % between pH 4–5 (Figure S1 and Table S1 in the Supporting Information). Aniline, a reported catalyst of oxime formation, did not appear to increase conjugation yields with fGly at any pH tested, and may have been inhibitory in this set of reactions.^37^ We obtained maximal protein labeling after 24 h at 37 °C (Figure S2 A in the Supporting Information). The reactions of hIgG with a peptide probe, aminooxy-FLAG (AO-FLAG),^32^ were dependent on the reagent concentration and required over ten equivalents (100 μM) of AO-FLAG for optimal labeling (Figure S2B in the Supporting Information). These results highlight the limitations of an exclusively oxime-based conjugation approach with sterically encumbered reactants, and also provided the impetus to explore Cu-free azide–alkyne cycloadditions for protein–protein assembly.^20^, ^38^

We generated three linkers of various lengths (**1**–**3**, Figure 1 B) that each contain an azide attached by a tetraethyleneglycol (TEG) spacer to an aminooxy moiety. For the cyclooctyne component, we chose the commercially available dibenzoazacyclooctyne (DIBAC).^30^, ^39^ Linkers **1**–**3** were treated with aldehyde-tagged MBP and subsequently an excess amount of the dibenzoazacyclooctyne fluorophore DIBAC-488. Robust labeling was observed by fluorescence gel scanning, which was dependent on the presence of the azide-functionalized linker (Figure 2). In contrast, direct labeling of MBP-fGly with AO-AF488 produced weaker labeling under similar conditions (Figure S3 in the Supporting Information). Furthermore, removal of excess azide linker before reaction with DIBAC-488 allowed the use of 15-fold less of the fluorophore reagent without affecting the yields (Figure 2). Linker **2**, which contains an aminooxy acetyl group, was the least efficient labeling reagent, as determined by MALDI-TOF MS analysis (Figure 2, see also Figure S4 in the Supporting Information). One concern was the possible side reactivity of DIBAC reagents with free thiols, which has been noted with other reactive cyclooctynes.^40^, ^41^ Our experiments with MBP could not address this issue, as the protein has no free cysteine residues. Thus, we performed a similar reaction with aldehyde-tagged human serum albumin (HSA), which contains a natural free cysteine residue. Treatment of aldehyde-tagged HSA with DIBAC-488 alone gave no significant labeling (Figure S5 in the Supporting Information). Thus, the low to sub-millimolar concentrations of DIBAC reagents that were used in our procedures do not appear to produce unwanted side reactions.^31^

**Figure 2:**
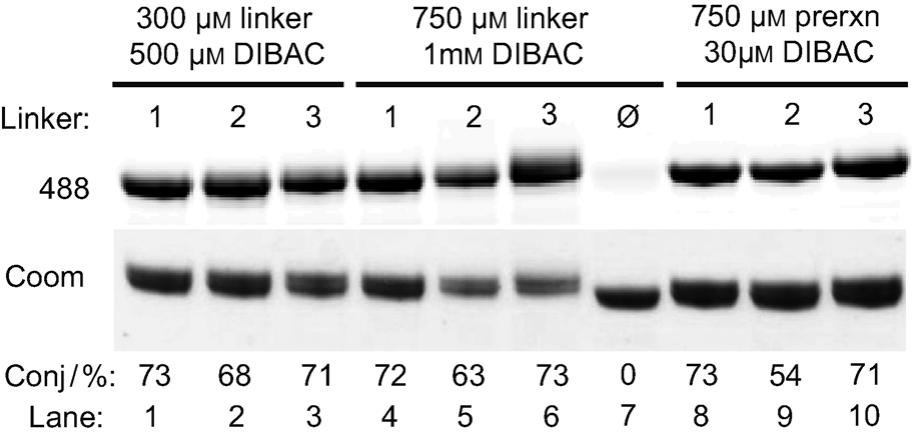
Reaction of fGly-containing MBP (30 μM) with bifunctional linkers **1**–**3** and subsequently with DIBAC-488. Lanes 1–6: protein treated with linkers (pH 4.5, 32 °C, 16 h) and then excess DIBAC-488 (16 h, 4 °C). Lane 7: fGly-MBP treated with DIBAC-488 alone, without prior linker conjugation. Lanes 8–10: linker was removed then azide-tagged MBP (15 μM) was treated with DIBAC-488 (2 equiv, 16 h, 4 °C). Top row: fluorescent scan; bottom row: coomassie stain.

As a next step, we explored protein–peptide conjugations by using a DIBAC-FLAG conjugate as a model peptide (see the Supporting Information). Aldehyde-tagged MBP was treated with linkers **1**–**3**, and the purified conjugate was coupled with DIBAC-FLAG. The Cu-free click reactions labeled MBP-fGly more efficiently than treatment with AO-FLAG alone, as demonstrated by immunoblot (Figure S6 in the Supporting Information). In more detailed comparisons, DIBAC-FLAG reactions were faster at room temperature than the corresponding AO-FLAG reactions at 37 °C and required lower reagent concentrations (Table S2 and Figure S7 in the Supporting Information).

To demonstrate the power of the Cu-free click chemistry approach, we generated conjugates of full-length hIgG with hGH^32^, ^34^ or MBP (Figure 3 A). These constructs are particularly relevant to ongoing efforts to increase the serum halflife of protein therapeutics (hGH-hIgG)^10^, ^42^ or to achieve dual binding specificities in a single molecule (MBP-hIgG).^5^, ^43^ Our strategy for fusing the protein pairs included the synthesis of bifunctional linker **4**, which comprises DIBAC tethered by a TEG spacer to an aminooxy group (Figure 1). We envisioned that an aldehyde-tagged protein could be treated with linker **1** and then combined with a protein that is conjugated to linker **4** to form a chemically and topologically defined protein homo or heterodimer.

**Figure 3:**
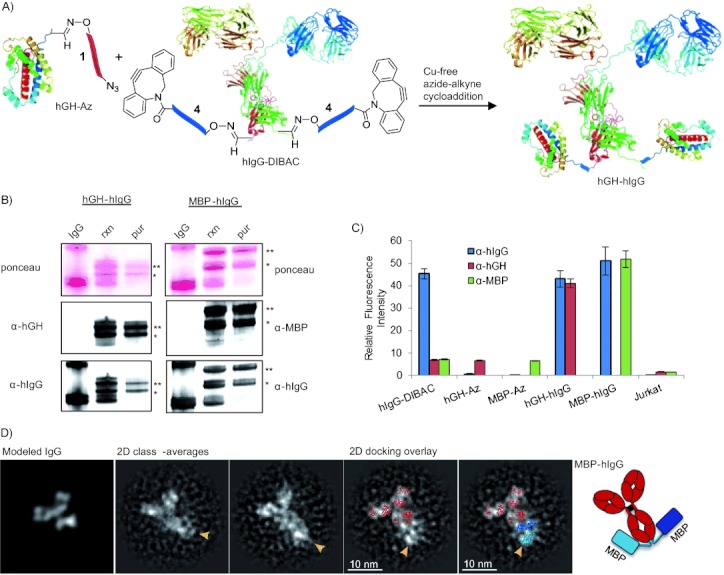
Protein–protein conjugation of hIgG with hGH and MBP. A) Aldehyde-tagged protein functionalized with azide **1** (hGH-Az) reacts specifically with protein functionalized with **4** (hIgG-DIBAC). As hIgG is a homodimer, two molecules of hGH-Az can react with hIgG-DIBAC to form a trimer. B) Western blot analysis of hIgG-hGH and hIgG-MBP chemical conjugations, nonreduced to highlight mono- and diconjugation. rxn: after reaction at 4 °C for 16 h. pur: after purification. Top blots: ponceau stain. Middle blots: blot probed with α-hGH or α-MBP and subsequently by α-mIgG HRP. Bottom blots: same blot probed with α-hIgG 647. * Denotes single conjugate; ** denotes diconjugate. C) Flow cytometry analysis of SKOV3 cells treated with aldehyde-tagged α-HER2-hIgG. Chemically conjugated hGH-hIgG and MBP-hIgG labeled the cell surface by α-HER2 binding, whereas azide-modified hGH-Az or MBP-Az alone did not. Blue=α-hlgG; red=α-hGH; green=α-MBP.D) Negative stain TEM image of C-terminal-tagged MBP-hIgG conjugates. A gallery of 2D class-averages of negatively stained MBP-hIgG shows a flexible additional density at the tip of one of the hIgG density lobes (arrows) that is consistent with a C-terminal attachment. The left panel displays a simulated density map of unconjugated IgG; the averages on the right are overlaid with a 2D docking of IgG1 alone (red) or with additional MBP crystal structures (light and dark blue; Protein Data Bank (PDB) 1IGY, 1JW4).

We treated linkers **1** or **4** with hGH, MBP, and hIgG separately, then subsequently treated the conjugates with DIBAC-488, azide Alexa Fluor 647 (Az-647), or the complementary Cu-free click protein partner. The oxime-conjugated proteins were efficiently labeled with dye and formed the expected homo and heterodimers (Figures S8 and S9 in the Supporting Information). Next, we established large-scale conjugation conditions for the reaction of azide-modified linker **1**-hGH or linker **1**-MBP with DIBAC-modified linker **4**-hIgG (2:1 azide protein/DIBAC protein, 4 °C, phosphate-buffered saline (PBS)). The resulting hIgG–protein chemical fusions (Figure 3 A) were purified and analyzed by immunoblot and transmission electron microscopy (TEM).

The hIgG construct used in this study has the aldehyde tag at the C termini of its two identical heavy chains. Thus, each fully assembled hIgG unit presents two sites for conjugation. As shown in Figure 3 B, the reactions of DIBAC-functionalized hIgG with azide-functionalized hGH or MBP produced two species with higher molecular weights in a nonreducing gel, which we attribute to the formation of mono and diconjugated proteins. Further confirmation of the product identities was obtained by immunoblot probing for hGH, MBP, and hIgG. Under reducing conditions, we detected the protein-conjugated hIgG heavy chain (Figure S10 in the Supporting Information). Over 70 % of hIgG was conjugated (over two steps; oxime formation and cycloaddition) according to densitometry analysis.

The generality of this approach to antibody–protein conjugation was assessed by generating similar fusions with a human antibody against the HER2/neu receptor, a common breast and ovarian cancer marker and target of the clinically approved antibody drug Herceptin.^44^ The anti-HER2/neu antibody was tagged with the aldehyde tag at the C terminus then conjugated to hGH and MBP by using the same protocol described for hIgG. We confirmed that the antibody–protein chemical conjugates retained antigen binding activity by using cell-based assays. The HER2-overexpressing cell line SKOV3 was incubated with the antibody–protein conjugates and analyzed by flow cytometry staining with anti-hGH, anti-MBP, and anti-hIgG antibodies. As shown in Figure 3 C and Figure S11 in the Supporting Information, the chemically conjugated antibody fully retained its ability to bind its target on SKOV3 cells and delivered its associated hGH or MBP domain to the cell surface. Importantly, the low-pH conditions of the initial oxime-forming reaction did not appear to impact antigen binding. No labelling was detected for azide-modified hGH-Az/MBP-Az alone or on Jurkat T cells, which do not express HER2.

As further proof of the structure of the conjugates, we performed a TEM analysis of the MBP-hIgG conjugate by using negative staining as well as single-particle alignment and classification. The resulting averaged 2D densities show characteristic three-lobed views of the IgG^45^ and a clear additional density that is comparable in size with one or two molecules of MBP at the end of one of the lobes, which is consistent with a C-terminal attachment. This was verified by 2D docking of IgG and MBP crystal structures to some of the class averages, as illustrated in Figure 3 D.

In conclusion, we have demonstrated that Cu-free click chemistry in conjunction with the aldehyde tag can produce protein–protein chemical conjugates of unprecedented size and complexity. The synthetic route capitalizes on small-molecule linkers that can increase reaction yields, lower the necessary reagent concentrations, and decrease the reaction time. The method should expand the topologies of available protein fusions and allow the exploration of alternate points of protein–protein attachment.

Possible applications in the antibody drug discovery space include antibody-dependent enzyme prodrug therapies (ADEPT) and antibody targeted immunotoxins.^46^–^48^ Furthermore, the approach can be extended to protein–synthetic polymer conjugations and surface immobilization^49^, ^50^ along with designing protein conjugates that extend serum halflife,^51^ or for vaccine development.^52^

## Experimental Section

General protein conjugation: A buffered solution (optimal pH 4.5) of aldehyde-tagged protein (10–50 μM) was treated with aminooxy reagent (0.2–1 mM, 10–20 equiv) and agitated at 35 °C for 16 h. Proteins were purified from low molecular weight reagents by buffer exchange or analyzed directly by SDS-PAGE. Subsequent Cu-free azide-alkyne cycloaddition reactions were conducted at 37 °C for 1 h or at 4 °C for 16 h in the case of protein–protein conjugations.

## Supporting Information

Detailed facts of importance to specialist readers are published as ”Supporting Information”. Such documents are peer-reviewed, but not copy-edited or typeset. They are made available as submitted by the authors.

## References

[1] Carter PJ (2011). Exp. Cell Res.

[2] Leader B, Baca QJ, Golan DE (2008). Nat. Rev. Drug Discovery.

[3] Schmidt SR (2009). Curr. Opin. Drug Discov. Devel.

[4] Chamow SM, Ashkenazi A (1999). Antibody Fusion Proteins.

[5] Beck A, Wurch T, Bailly C, Corvaia N (2010). Nat. Rev. Immunol.

[6] Gillies S, Lan Y, Brunkhorst B, Wong W-K, Li Y, Lo K-M (2002). Cancer Immunol. Immunother.

[7] Cochran JR (2010). Sci. Transl. Med.

[8] Ho VT, Zahrieh D, Hochberg E, Micale E, Levin J, Reynolds C, Steckel S, Cutler C, Fisher DC, Lee SJ (2004). Blood.

[9] Filpula D (2007). Biomol. Eng.

[10] Wilkinson IR, Ferrandis E, Artymiuk PJ, Teillot M, Soulard C, Touvay C, Pradhananga SL, Justice S, Wu Z, Leung KC (2007). Nat. Med.

[11] Huang C (2009). Curr. Opin. Biotechnol.

[12] Walsh G (2010). Nat. Biotechnol.

[13] Baggio LL, Huang Q, Brown TJ, Drucker DJ (2004). Diabetes.

[14] Teillaud J-L (2005). Expert Opin. Biol. Ther.

[15] Chames P, Baty D (2009). mAbs.

[16] Xiao J, Tolbert TJ (2009). Org. Lett.

[17] McGinty RK, Kim J, Chatterjee C, Roeder RG, Muir TW (2008). Nature.

[18] Sommer S, Weikart ND, Brockmeyer A, Janning P, Mootz HD Angew. Chem.

[19] Chalker JM, Bernardes GJL, Davis BG (2011). Acc. Chem. Res.

[20] Lim RKV, Lin Q (2010). Chem. Commun.

[21] Hutchins BM, Kazane SA, Staflin K, Forsyth JS, Felding-Habermann B, Smider VV, Schultz PG (2011). Chem. Biol.

[22] Bundy BC, Swartz JR (2010). Bioconjugate Chem.

[23] Jewett JC, Bertozzi CR (2010). Chem. Soc. Rev.

[24] Lallana E, Riguera R, Fernandez-Megia E Angew. Chem.

[25] Sletten EM, Bertozzi CR (2011). Acc. Chem. Res.

[26] Debets MF, van Berkel SS, Dommerholt J, Dirks AJ, Rutjes FPJT, van Delft FL (2011). Acc. Chem. Res.

[27] Laughlin ST, Baskin JM, Amacher SL, Bertozzi CR (2008). Science.

[28] Hur GH, Meier JL, Baskin J, Codelli JA, Bertozzi CR, Marahiel MA, Burkart MD (2009). Chem. Biol.

[29] Mbua NE, Guo J, Wolfert MA, Steet R, Boons G-J (2011). ChemBioChem.

[30] Debets MF, van Berkel SS, Schoffelen S, Rutjes FPJT, van Hest JCM, van Delft FL (2010). Chem. Commun.

[31] Jewett JC, Sletten EM, Bertozzi CR (2010). J. Am. Chem. Soc.

[32] Carrico IS, Carlson BL, Bertozzi CR (2007). Nat. Chem. Biol.

[33] Wu P, Shui W, Carlson BL, Hu N, Rabuka D, Lee J, Bertozzi CR (2009). Proc. Natl. Acad. Sci. USA.

[34] Hudak JE, Yu HH, Bertozzi CR (2011). J. Am. Chem. Soc.

[35] Dierks T, Dickmanns A, Preusser-Kunze A, Schmidt B, Mariappan M, von Figura K, Ficner R, Rudolph MG (2005). Cell.

[36] Rabuka D (2010). Curr. Opin. Chem. Biol.

[37] Dirksen A, Dawson PE (2008). Bioconjugate Chem.

[38] Sletten EM, Bertozzi CR Angew. Chem.

[39] Kuzmin A, Poloukhtine A, Wolfert MA, Popik VV (2010). Bioconjugate Chem.

[40] Conte ML, Staderini S, Marra A, Sanchez-Navarro M, Davis BG, Dondoni A (2011). Chem. Commun.

[41] Fairbanks BD, Sims EA, Anseth KS, Bowman CN (2010). Macromolecules.

[42] Osborn BL, Sekut L, Corcoran M, Poortman C, Sturm B, Chen G, Mather D, Lin HL, Parry TJ (2002). Eur. J. Pharmacol.

[43] Caravella J, Lugovskoy A (2010). Curr. Opin. Chem. Biol.

[44] Ménard S, Pupa SM, Campiglio M, Tagliabue E (2003). Oncogene.

[45] Roux KH (1999). Int. Arch. Allergy Immunol.

[46] Schellmann N, Deckert PM, Bachran D, Fuchs H, Bachran C (2010). Mini-Rev. Med. Chem.

[47] Andrady C, Sharma SK, Chester KA (2011). Immunotherapy.

[48] Pastan I, Hassan R, FitzGerald DJ, Kreitman RJ (2006). Nat. Rev. Cancer.

[49] Le Droumaguet B, Velonia K (2008). Macromol. Rapid Commun.

[50] Lin P-C, Ueng S-H, Tseng M-C, Ko J-L, Huang K-T, Yu S-C, Adak AK, Chen Y-J, Lin C-C Angew. Chem.

[51] Roland K (2011). Curr. Opin. Biotechnol.

[52] Timmerman JM (2009). Hum. Vaccines.

