# Supplementary material for: Synthesis of Heterobifunctional Protein Fusions Using Copper-Free Click Chemistry and the Aldehyde Tag
Source: Angew Chem Int Ed Engl. 2012 Mar 12;51(17):4161–5. doi: 10.1002/anie.201108130 (PMC3379715; doi:10.1002/anie.201108130)
Supplement: Supplementary file 1 [file anie0051-4161-SD1.pdf]

Angewandte Chemie
Eine Zeitschrift der Gesellschaft Deutscher Chemiker

Supporting Information

© Wiley-VCH 2012

69451 Weinheim, Germany

Synthesis of Heterobifunctional Protein Fusions Using Copper-Free Click Chemistry and the Aldehyde Tag**

Jason E. Hudak, Robyn M. Barfield, Gregory W. de Hart, Patricia Grob, Eva Nogales, Carolyn R. Bertozzi, and David Rabuka**

anie_201108130_sm_miscellaneous_information.pdf

Supporting Information

Table of Contents

I. **Supporting Figures**

II. **Materials and Methods**

a. Chemical synthesis

b. Expression and purification of aldehyde-tagged proteins

c. Oxime formation and optimization

d. Linker and oxime vs. Cu-free click conjugations

e. Protein-protein conjugations, flow cytometry, and TEM

III. **Supporting References**

I. Supporting Figures

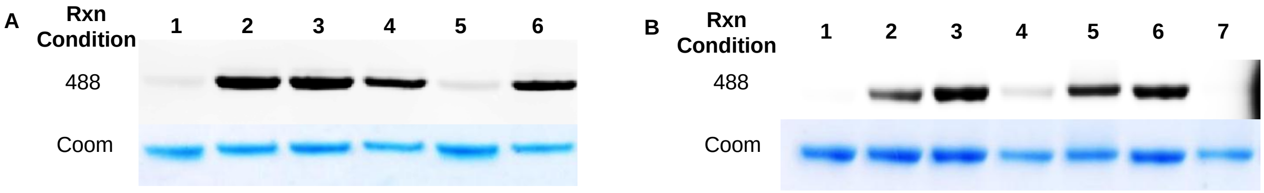

Figure S1. Increasing yield of conjugation with AO-AF488 at decreasing pH. (A) SDS-PAGE analysis of 25 µM MBP reacted with 400 µM AO-AF488 (37 ºC, 18h). (B) SDS-PAGE analysis of 20 µM hIgG reacted with 500 µM AO-AF488 (37 ºC, 18h). Reaction conditions correspond to those in Table **S1**, below**.** An aliquot was retained for PAGE analysis and the remaining portion was buffer exchanged for quantitative analysis by UV absorbance (Table **S1**, below). Top, fluorescent scan; bottom, coomassie stain.

Table S1. Conjugation yields with AO-AF488 under various buffer and pH conditions

Protein[a]	Rxn Condition	Buffer, pH [b]	DOL (%)[c]	Protein[a]	Rxn Condition	Buffer, pH [b]	DOL (%)[c]
**MBP-Ald**	1	PBS, 7	8	**hIgG-Ald**	1	PBS, 7	12
	2	MES, 5.5	35		2	MES, 5.5	48
	3	KOAc, 4.6	46		3	KOAc, 4.6	70
	4	KOAc/aniline, 4.6	28		4	KOAc/aniline, 4.6	33
	5	5% MeCN, ~8	9		5	NaCit, 3.5	73
	6	MeCN/AcOH, 5	52		6	MeCN/AcOH, 5	76
					7	KOAc/$MeONH_2$, 4.6	7

[a] 25 µM MBP, 400 µM AO-AF488; or 20 µM hIgG, 500 µM AO-AF488 as in Figure S1 [b] All buffer components at 100 mM except PBS and aniline (10 mM), MeCN (5%), and AcOH (.02%) [c] Degree of labelling (DOL) determined by corrected absorbance ratio (280/494) after buffer exchange by concentration in to PBS.

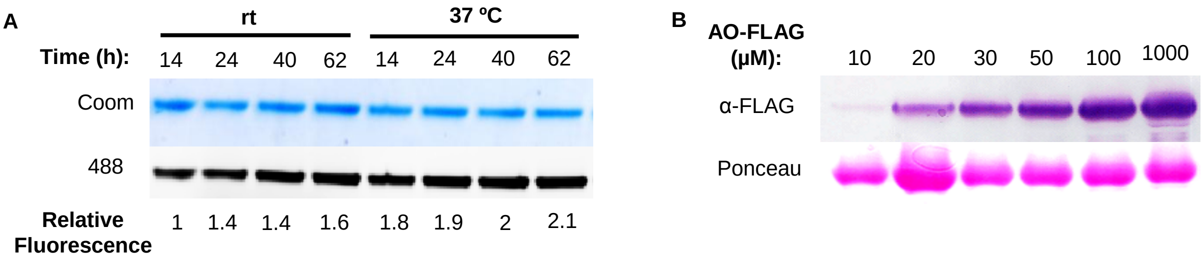

Figure S2. Assessment of oxime formation with hIgG at varying times, temperatures, and concentrations. (A) 5 µM hIgG was reacted with 200 µM AO-CF488 (KOAc, pH 4.5). Top, coomassie stain; bottom, AO-CF488 fluorescence. (B) 10 µM hIgG was reacted with AO-FLAG (rt, 16 h, 5% MeCN/0.02% FA). Top, blot probed with mouse α-FLAG and goat α-mIgG-AP conjugate; bottom, ponceau.

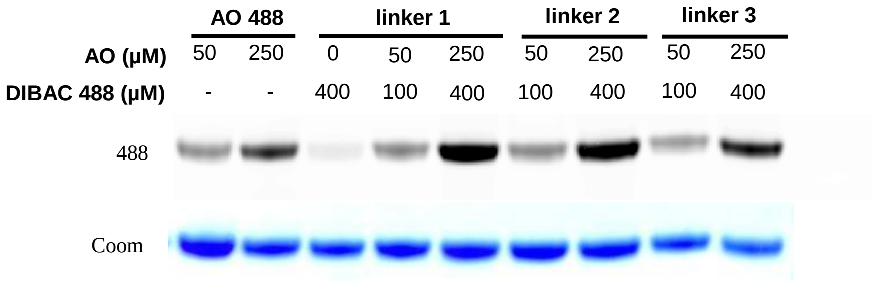

Figure S3. Linkers functionalize aldehyde-tagged hIgG with azide for subsequent Cu-free click chemistry. SDS-PAGE analysis of 5 µM hIgG reacted with AO-AF488 or AO linkers **1-3** (pH 4.5, RT, 18h) followed by DIBAC-488 (37 ºC, 1h). Top, fluorescent scan; bottom, coomassie stain.

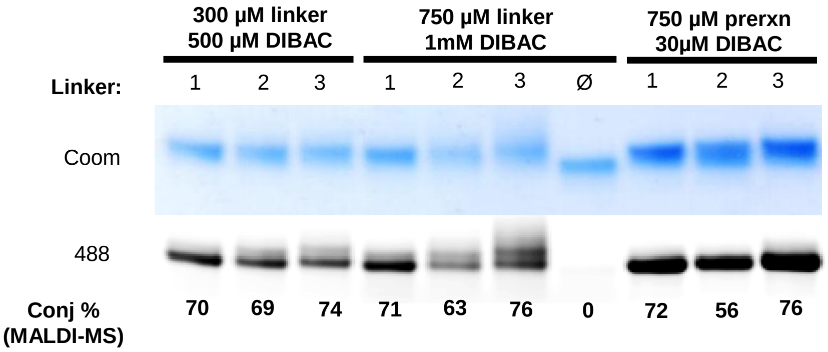

Figure S4. SDS-PAGE and MALDI-TOF MS analysis of reactions with DIBAC-488. 30 µM hGH was reacted with DIBAC-488 after conjugation with increasing concentrations of linkers **1-3**. Lanes 1-6, protein was reacted overnight with aminooxy linker in NaH_2PO_4/NaCit buffer pH 4.5 at 32 ºC followed by the addition of excess DIBAC-488 and reacted for an additional 16 h at 4º C. Lane 7, negative control reaction with only DIBAC-488 without linker conjugation. Lanes 8-10, conjugated protein was buffer exchanged to remove linker and then 15 µM of the azide tagged protein was reacted with only 2 equiv. of DIBAC-488 again for 16 h at 4º C. Aliquots were taken and run on SDS-PAGE or diluted with matrix solution for MALDI-TOF MS analysis.

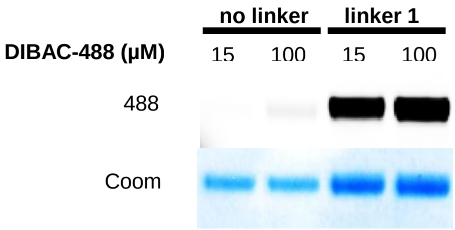

Figure S5. SDS-PAGE of aldehyde-tagged human serum albumin (HSA) reacted with DIBAC-488 with and without azide linker **1** conjugation. HSA was reacted at 25 µM with 1 mM linker **1** (pH 4.5, 35 ºC, 16h) and buffer exchanged into PBS. 5 µM of aldehyde- or azide(**1**)-tagged HSA was reacted with DIBAC-488 (pH 7.4, 4 ºC, 20h). While robust labeling was seen with the linker **1** conjugated protein (lanes 3,4), little to no reactivity was found for the HSA containing no azide but still a free thiol (lanes 1,2). Top, fluorescent scan; bottom, coomassie stain.

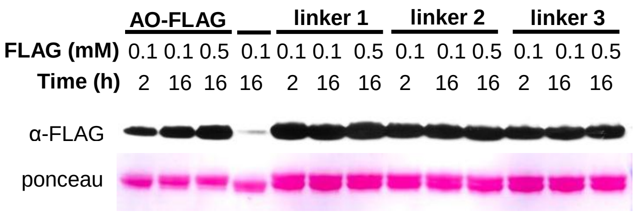

Figure S6. Western blot of 10 µM MBP reacted with 0.1-0.5 mM AO-FLAG (37 ºC, 2-16h) or 1 mM AO linkers **1-3** (37 ºC, 16h) followed by DIBAC-FLAG (rt, 2-16h) after linker removal. Lane 4 is incubated with DIBAC-FLAG but no linker showing minimal background. Top, blot probed with α-FLAG HRP; bottom, ponceau stain.

Table S2. Comparison of AO-FLAG vs. DIBAC-FLAG reaction yields over 24 h

Protein (20 µM)[a]	Concentration[b]	reagent	Yield (%, MALDI-MS[c]) 2 h	4 h	6 h	24 h
MBP-Ald	40 µM	AO-FLAG	7	12	17	32
MBP-N_3		DIBAC-FLAG	32	41	44	54
MBP-Ald	400 µM	AO-FLAG	28	47	48	69
MBP-N_3		DIBAC-FLAG	52	61	62	71
hGH-Ald	40 µM	AO-FLAG	11	21	31	53
hGH-N_3		DIBAC-FLAG	25	35	43	66
hGH-Ald	400 µM	AO-FLAG	43	54	59	69
hGH-N_3		DIBAC-FLAG	68	69	71	72

[a] 20 µM aldehyde-tagged protein (hGH-Ald or MBP-Ald) was reacted with 40 or 400 µM AO-FLAG in MES buffer pH 4.6 at 37 ºC. Linker **1** azide labelled protein (hGH-N_3 and MBP-N_3) was generated by reaction with linker **1** and buffer exchanged to remove excess linker before reaction with DIBAC-FLAG in PBS at rt. [b] Reactions were run in parallel at 2 or 20-fold FLAG labelled reaction partner and aliquots taken at specified times [c] Representative MALDI-TOF spectra of hGH-Ald vs. hGH-N_3 reactions with 40 µM FLAG are shown in Figure S4.

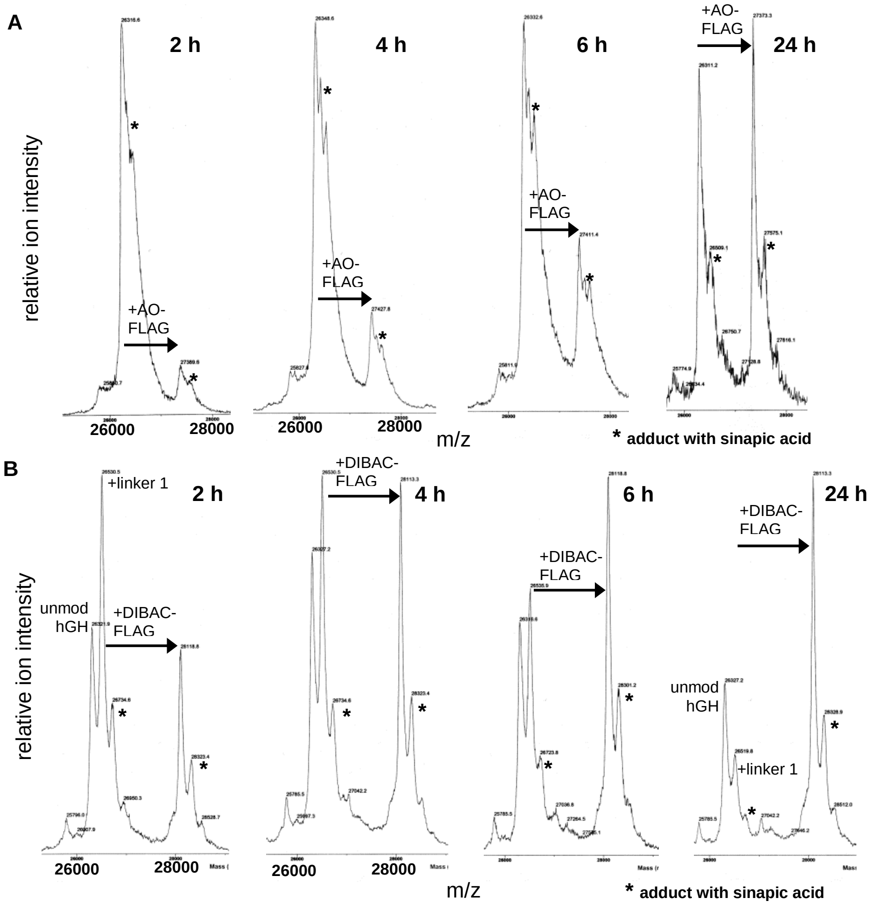

Figure S7. Representative MALDI-TOF MS analysis of hGH conjugation with 40 µM AO-FLAG or DIBAC-FLAG (after linker **1** conjugation) over 24 h. Arrows indicate mass shifts corresponding to the addition of the FLAG peptide epitope. Adducts with the sinapic acid matrix were observed and marked as * though did not alter conjugation analysis.

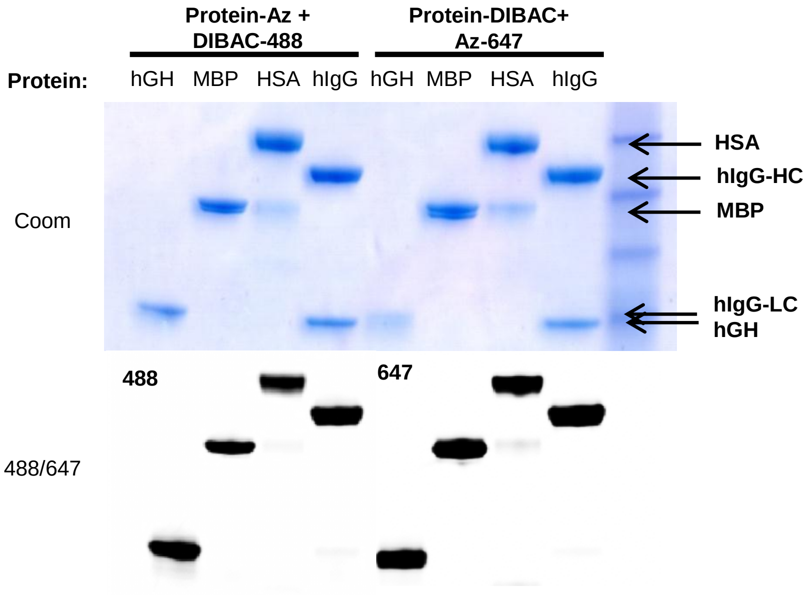

Figure S8. SDS-PAGE analysis of conjugation with the corresponding fluorescent Cu-free azide-alkyne click partner after reaction with linkers **1** or **4**. 25-50 µM aldehyde-tagged protein was reacted with 750 µM of linker **1** or **4** in NaH_2PO_4/NaCit buffer pH 4.5 at 35 ºC for 18 h. After buffer exchange into PBS, Cu-free click reactions were performed at 4 ºC, 16 h and run on SDS-PAGE. Lanes 1-3, 10 µM protein-Az (reacted with linker **1**) and 20 µM DIBAC-488; lanes 4-6, 10 µM protein-DIBAC and 20 µM Az-647; Top, coomassie stain; bottom, fluorescent scan at 488 nm for DIBAC-488 or 647 nm for Az-647. Human serum albumin (HSA), antibody heavy chain (hIgG-HC, contains aldehyde tag), antibody light chain (hIgG-LC), maltose binding protein (MBP), human growth hormone (hGH).

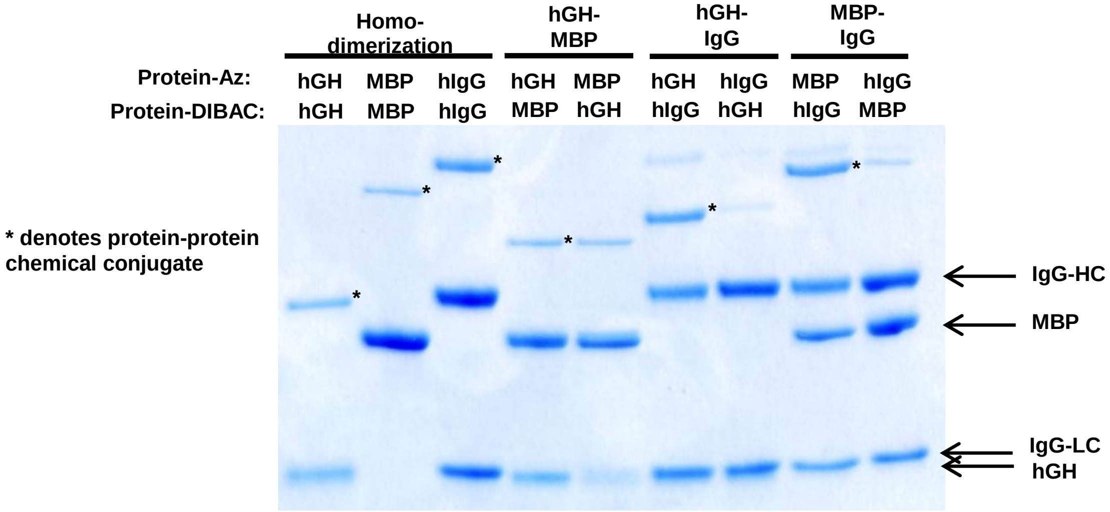

Figure S9. SDS-PAGE analysis of conjugation with the corresponding protein Cu-free click partner after reaction with linkers **1** or **4**. 25-50 µM aldehyde-tagged protein was reacted with 750 µM of linker **1** or **4** in NaH_2PO_4/NaCit buffer pH 4.5 at 35 ºC for 18 h. After buffer exchange into PBS, Cu-free click reactions were performed with 5 µM each protein pair at 4 ºC, 16 h and run on SDS-PAGE. Lanes 1-3, protein-Az reacted with protein-DIBAC of identical protein (5 µM each); lanes 4-9, 5 µM protein-Az reacted with 5 µM protein-DIBAC. Human serum albumin (HSA), antibody heavy chain (hIgG-HC, contains aldehyde tag), antibody light chain (hIgG-LC), maltose binding protein (MBP), human growth hormone (hGH).

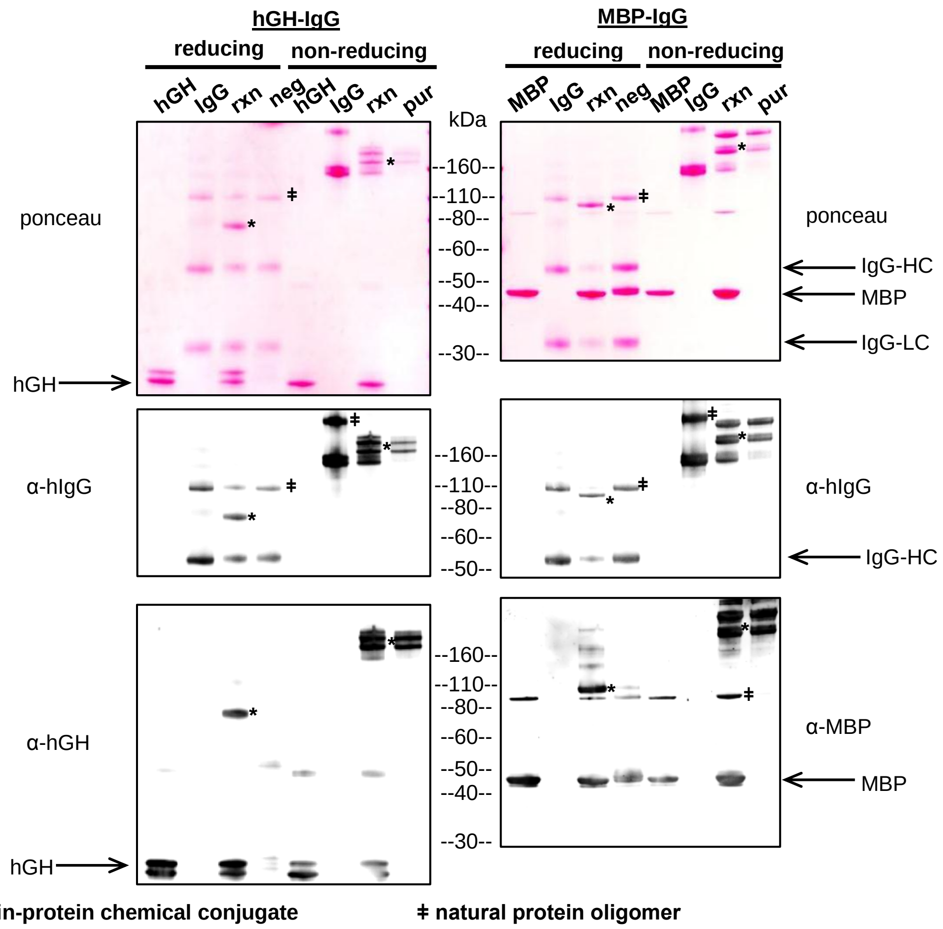

Figure S10. Western blot analysis of Cu-free click conjugations of hGH-Az and MBP-Az (linker **1**) with hIgG-DIBAC (linker **4**). Lanes 1-4, samples were reduced with βME to show specific labeling of the IgG heavy chain (IgG-HC); lane 4 includes reverse conjugate where linkers **1** and **4** were pre-click reacted and incubated with aldehyde tagged proteins to demonstarte lack of conjugation based on oxime formation alone; lanes 5-8, illustrate full-length conjugates containing one or two hGH/MBP molecules per hIgG dimer. Top, ponceau stain; middle, blot probed with α-hIgG 647 and imaged by fluorescent scan; bottom, same blot probed with α-hGH or α-MBP and imaged via α-mIgG-FITC to highlight higher MW conjugates containing the hGH or MBP conjugates.

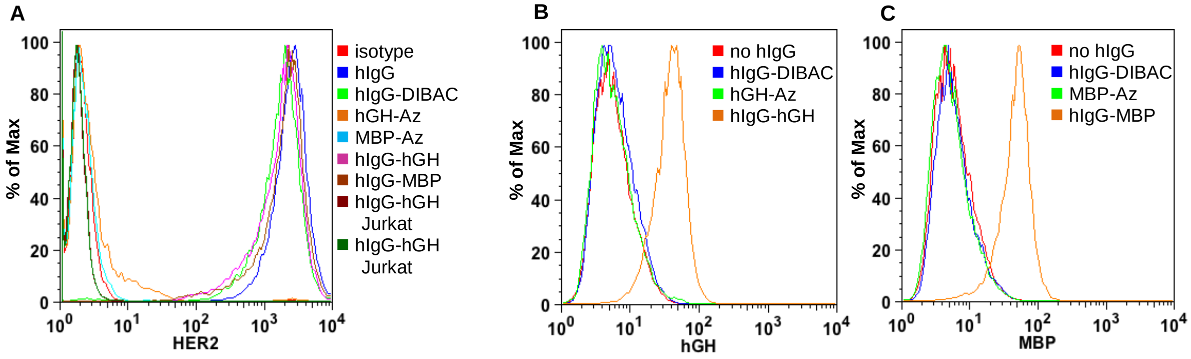

Figure S11. Representative histograms of fluorescence (x-axis) vs % of total cell counts (y-axis) for SKOV3 cells incubated with aldehyde tagged α-HER2 hIgG without and with conjugation to hGH/MBP. Cells were treated with a 1/500 dilution of 5 μM click protein-protein conjugation reactions followed by α-hIgG-649 to label the aldehyde-tagged α-HER2 hIgG and mouse α-hGH or α-MBP followed by α-mIgG to label the presence of hGH/MBP. (A) 647 channel showing binding of the conjugated and nonconjugated α-HER2 hIgG to SKOV3 cells but not Jurkat T cells. No appreciable loss in binding is observed for the hIgG conjugate. (B) 488 channel demonstrates delivery of hGH to cells only with the hIgG-hGH chemical conjugations. (C) 488 channel for MBP labelling.

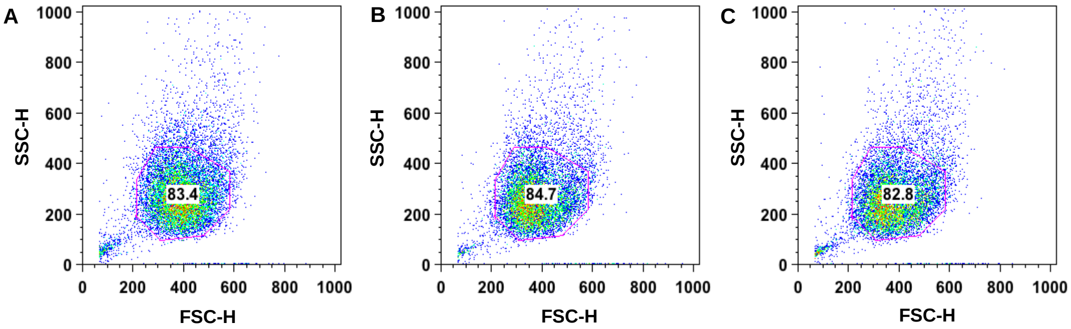

Figure S11. Representative dot-plots of forward scatter (x-axis) vs side scatter (y-axis) for SKOV3 cells incubated (A) without aldehyde tagged α-HER2 hIgG conjugates and with (B) hIgG-hGH and (C) hIgG-MBP conjugates. No apparent abnormalities or gross cell death was observed throughout the sample set.

II. Materials and Methods

a. Chemical synthesis

General materials

Synthetic reagents were purchased from Sigma-Aldrich, Acros, and TCI and used without purification unless noted otherwise. DIBAC-488, DIBAC-NHS ester, and sulfo-DIBAC-NHS ester were purchased from Click Chemistry Tools (Scottsdale, AZ). Aminooxy-FLAG peptide containing an N-terminal aminooxy acetic acid[1] was custom synthesized by New England Peptide (Gardner, MA). Linkers **2** and **3** were custom synthesized by Tandem Sciences Inc. (Menlo Park, CA). Anhydrous DMF and MeOH were purchased from Acros in sealed bottles; all other anhydrous solvents were obtained from an alumina column solvent purification system. All reactions were carried out in flame-dried glassware under N_2 unless otherwise noted. In all cases, solvent was removed by reduced pressure with a Buchi Rotovapor R-114 equipped with a Welch self-cleaning dry vacuum. Products were further dried by reduced pressure with an Edwards RV3 high vacuum. Lyophilization was performed on a LABCONCO FreeZone instrument equipped with an Edwards RV2 pump. Thin layer chromatography was performed with Silicycle 60 Å silica gel plates and detected by UV lamp or charring with *p*-anisaldehyde in acidic EtOH. Flash chromatography was performed using Silicycle 60 Å 230-400 mesh silica. HPLC was performed on a Varian system attached to a absorption detector using a C18 reverse phase column (5 µm, 250 x 4.6 mm, Agilent(Varian); Carlsbad, CA) for analytical or a C18 reverse phase column (8 µm, 250 x 21.4 mm, Agilent(Varian); Carlsbad, CA) for preparative purifications. HPLC solvents were A: ddH_2O with 0.1% TFA and B: MeCN with 0.1% TFA. All ^{1}H and ^{13}C NMR spectra are reported in ppm and referenced to solvent peaks (1H and 13C). NMR spectra were obtained on Bruker AVQ-400, DRX-500, AV-500, or AV-600 instruments. High resolution electrospray ionization (ESI) mass spectra were obtained from the UC Berkeley Mass Spectrometry Facility an LTQ Orbitrap (Thermo Fisher Scientific). MALDI-TOF (Matrix-assisted laser desorption/ionization (time-of-flight)) analysis was carried out on an Applied Biosystems Voyager DE-Pro machine.

*Synthetic procedures and analytical data for linkers **1-4** and DIBAC-FLAG*

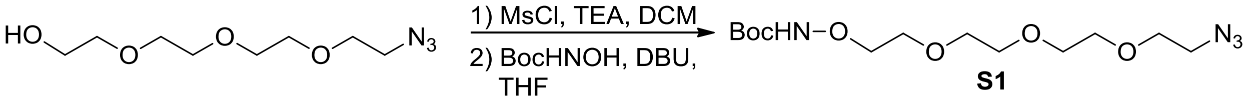

***Tert*-butyl 2-(2-(2-(2-azidoethoxy)ethoxy)ethoxy)ethoxycarbamate (S1):** To known 2-(2-(2-(2-Azidoethoxy)ethoxy)ethoxy)ethanol[2] (581 mg, 2.65 mmol) in CH_2Cl_2 (4 mL) was added triethylamine (TEA, 554 µL, 3.98 mmol) at 4 ºC. The solution was maintained at 4 ºC upon which MsCl (308 µL, 3.98 mmol) was added dropwise and allowed to warm to rt over 16 h. Upon completion, the reaction was quenched with 2M aqueous NH_4Cl, extracted with CH_2Cl_2, and the organic layer washed with brine and dried over Na_2SO_4. The concentrated residue along with BocHNOH (494 mg, 3.71 mmol) was dissolved in Et_2O (3 mL) and DBU (594 µL, 3.98 mmol) was added dropwise at 4 ºC. The reaction was allowed to warm to rt and stirred for 3 days. CH_2Cl_2 was added and the organic layer was washed with 2M aqueous NH_4Cl, brine, and dried over Na_2SO_4. The concentrated residue was purified by silica flash chromatography (Hex/EtOAc gradient) to obtain protected aminooxy TEG linker **S1** as a clear oil (361 mg, 41%). Data was consistent with previous reported values[3]: R_f = 0.5 (1:3, Hex/EtOAc); ^{1}H NMR (400 MHz, $CDCl_3$) δ 7.85 (s, 1H), 3.88 (dd, *J* = 5.5, 3.8 Hz, 2H), 3.60 – 3.52 (m, 12H), 3.28 – 3.23 (m, 2H), 1.35 (s, 9H).

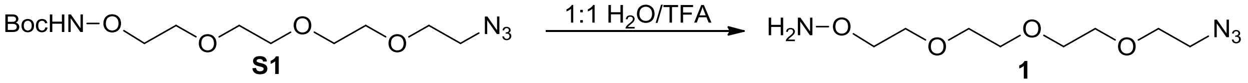

***O*-(2-(2-(2-(2-azidoethoxy)ethoxy)ethoxy)ethyl)hydroxylamine (1):** Protected aminooxy linker **S1** (115 mg, 0.344 mmol) was dissolved in H_2O/TFA (1:1; v:v; 3 mL) and stirred at rt for 1.5 h. The reaction was monitored by analytical reverse phase HPLC and upon completion was concentrated and redissolved in ddH_2O for preparative HPLC purification. The product eluted at 10 min in a gradient of 5% to 75% MeCN in ddH_2O (0.1% TFA) over 30 min. The product peaks were collected and lyophilized to obtain aminooxy azide linker **1** as a clear oil (80 mg, 99%): ^{1}H NMR (600 MHz, D_2O) δ 4.20 (app t, 2H), 3.78 (app t, 2H), 3.68 – 3.65 (m, 11H), 3.44 (app t, 2H); ^{13}C NMR (151 MHz, D_2O) δ 73.83, 69.76, 69.49, 69.42, 69.38, 69.11, 68.29, 50.09; HRMS (ESI): calcd for $C_8H_{18}N_4O_4$ $[M+H]^+$ *m/z* = 235.1401, found: 235.1399.

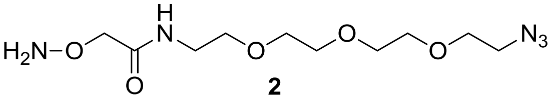

2-(aminooxy)-*N*-(2-(2-(2-(2-azidoethoxy)ethoxy)ethoxy)ethyl)acetamide (2): Aminooxy acetic acid azide linker **2** was prepared by custom synthesis by Tandem Sciences Inc. following modified procedures[4]: ^{1}H NMR (600 MHz, D_2O) δ 4.62 (s, 2H), 3.73 – 3.66 (m, 10H), 3.65 (t, *J* = 5.3 Hz, 2H), 3.47 (dt, *J* = 10.7, 5.1 Hz, 4H); ^{13}C NMR (151 MHz, D_2O) δ 168.90, 71.83, 69.63, 69.61, 69.56, 69.46, 69.22, 68.65, 50.23, 38.77; HRMS (ESI): calcd for $C_{10}H_{22}N_5O_5$ $[M+H]^+$ *m/z* = 292.1615, found: 292.1623.

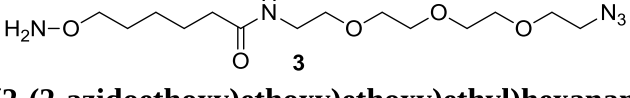

6-(aminooxy)-*N*-(2-(2-(2-(2-azidoethoxy)ethoxy)ethoxy)ethyl)hexanamide (3): Aminooxy hexanoic acid azide linker **3** was custom sythesized by Tandem Sciences Inc. following modified

procedures[4]: ^{1}H NMR (600 MHz, D_2O) δ 3.77 – 3.67 (m, 12H), 3.62 (t, J = 5.3 Hz, 2H), 3.50 (t, J = 5.3 Hz, 2H), 3.38 (t, J = 5.3 Hz, 2H), 2.26 (t, J = 7.4 Hz, 2H), 1.60 (qd, J = 14.7, 7.1 Hz, 4H), 1.34 (dq, J = 15.3, 7.8 Hz, 2H); ^{13}C NMR (151 MHz, D_2O) δ 176.94, 75.87, 69.64, 69.59, 69.46, 69.25, 68.88, 50.19, 38.89, 35.63, 27.13, 25.15, 24.65; HRMS (ESI): calcd for $C_{14}H_{30}N_5O_5$ $[M+H]^+$ m/z = 348.2241, found: 348.2249.

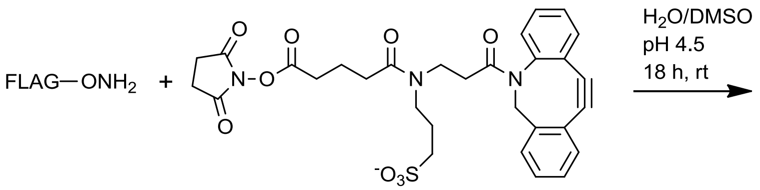

Sulfo-dibenzoazacyclooctyne-FLAG conjugate (DIBAC-FLAG): Aminooxy-FLAG [H_2NO-CH_2-CO-NH-(DYKDDDDK)-CO_2H] (1.2 mg, .0011 mmol) was dissolved in KOAc buffer (50 mM, pH 4.5, 420 µL). To this was added a 10 mg/mL stock of sulfo-DIBAC-NHS ester (79 µL, .0013 mmol) and rotated for 18 h at rt. The resulting product mixture was purified directly by analytical HPLC in a gradient of 20% to 50% MeCN in ddH_2O (0.1% TFA) over 30 min with elution at 16 min. The product peaks were collected and lyophilized to obtain DIBAC-FLAG as a white powder (0.2mg, 13%). Separation from unconjugated AO-FLAG and diconjugated DIBAC-FLAG was confirmed by western blot (not shown): HRMS (ESI): calcd for $C_{69}H_{85}N_{13}O_{28}S_1$ $[M+2H]^{2+}$ m/z = 788.7758, found: 788.7744.

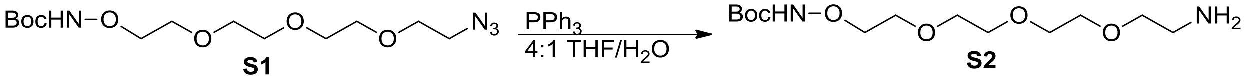

2-[2-(2-*N*-(*tert*-Butyloxycarbonyl)aminooxyethoxy)-ethoxy]ethylamine (S2): Protected aminooxy linker **S1** (150 mg, 0.344 mmol) was dissolved in THF/H_2O (4:1; v:v; 4 mL) and stirred at rt for 1.5 h. At this point, 1 M NaOH (10 µL) was added and allowed to stir at rt for 20 h. The crude solution was concentrated and purified by silica flash chromatography (EtOAc/MeOH/H_2O gradient) to obtain protected aminooxy amine linker **S2** as a yellow oil (116 mg, 84%). Data was consistent with previous reported values[5]: R_f = 0.33 (4:2:1, EtOAc/MeOH/H_2O); ^{1}H NMR (500 MHz, $CDCl_3$) δ 4.01 – 3.95 (m, 2H), 3.71 – 3.66 (m, 2H), 3.66 – 3.59 (m, 10H), 3.50 (t, J = 5.2 Hz, 2H), 2.86 – 2.80 (m, 2H), 1.44 (d, J = 3.1 Hz, 9H); ^{13}C NMR (126 MHz, $CDCl_3$) δ 157.01, 81.31, 75.33, 73.12, 70.67, 70.64, 70.59, 70.28, 69.23, 41.62, 28.34.

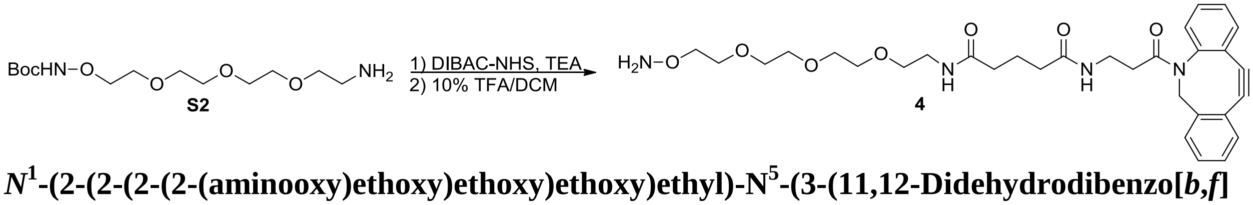

***N*^{1}-(2-(2-(2-(2-(aminooxy)ethoxy)ethoxy)ethoxy)ethyl)-N^5-(3-(11,12-Didehydrodibenzo[*b*,*f*] azocin-5(6*H*)-yl)-3-oxopropyl)glutaramide (4):** Protected aminooxy linker **S2** (5.0 mg, 0.016 mmol) and DIBAC-NHS ester (5.2 mg, 0.011 mmol) were dissolved in CH_2Cl_2 (500 µL) and TEA (2.2 µL, 0.016 mmol) was added dropwise at 4 ºC. The reaction was allowed to warm to rt over 16 h and monitored by TLC. The crude mixture was concentrated under vacuum and purified by silica flash chromatography (MeOH/DCM gradient) to obtain the protected aminooxy DIBAC intermediate linker as a yellow oil (5.2 mg, 70%); HRMS (ESI): calcd for $C_{36}H_{48}N_4O_9$ $[M+Na]^+$ m/z = 703.3314, found: 703.3303. The protected linker (5.2 mg, 0.0076

mmol) was dissolved in TFA/DCM (1:9; v/v; 300 µL) and stirred at rt for 45 min. The reaction was monitored by TLC (10% MeOH/DCM) and upon completion was concentrated, redissolved in 15% MeCN/ddH_2O, and filtered for HPLC purification. The product eluted near 50% MeCN in a gradient of 15% to 65% MeCN in ddH_2O (0.1% TFA) over 25 min. The product peaks were collected and lyophilized to obtain aminooxy-DIBAC linker **4** as a clear oil (2.6 mg, 60%): R_f = 0.55 (1:9, MeOH/CH_2Cl_2); ^{1}H NMR (500 MHz, D_2O) δ 7.67 (d, J = 7.4 Hz, 1H), 7.55 – 7.41 (m, 5H), 7.39 (t, J = 7.6 Hz, 1H), 7.33 (app d, J = 7.3 Hz, 1H), 5.09 (d, J = 14.2 Hz, 1H), 4.25 – 4.18 (m, 2H), 3.85 – 3.75 (m, 3H), 3.71 – 3.61 (m, 10H), 3.39 (t, J = 5.4 Hz, 2H), 3.18 – 3.11 (m, 1H), 3.10 – 3.02 (m, 1H), 2.35 – 2.30 (m, 1H), 2.26 – 2.15 (m, 1H), 2.09 (t, J = 7.5 Hz, 2H), 2.02 (dd, J = 14.7, 7.3 Hz, 2H), 1.72 – 1.60 (m, 2H); ^{13}C NMR (151 MHz, D_2O) δ 175.86, 175.37, 173.89, 131.95, 129.21, 129.12, 128.96, 128.54, 128.18, 127.12, 125.81, 122.42, 121.55, 99.95, 73.89, 69.78, 69.63, 69.44, 69.40, 68.87, 68.30, 55.47, 38.96, 35.81, 34.92, 34.85, 33.82, 21.66; HRMS (ESI): calcd for $C_{31}H_{41}N_4O_7$ $[M+H]^+$ m/z = 581.2970, found: 581.2966.

b. Expression and purification of aldehyde-tagged proteins

Recombinant proteins produced in *E. coli* (MBP and hGH) were expressed as previously described.[1] In short, BL21(DE3) *E. coli* harboring both a pET plasmid containing the aldehyde tagged-protein and a pBAD plasmid containing the *M. tuberculosis* FGE were grown in LB media supplemented with 100 µg/mL ampicillin and 50 µg/mL kanamycin at 37º C. When OD_{600} reached 0.5, arabinose was added at 0.25% and the culture was shaken at 37º C for an additional 1 h. The temperature was then reduced to 18º C for 1 h and IPTG (0.1 mM) was added with further shaking for 14-18 h. Cells were lysed by homogenization, and the His_6-tagged proteins were purified using Ni-NTA-agarose beads (Qiagen) under standard purification procedures. MBP was eluted in 50 mM NaH_2PO_4, 300 mM NaCl, 250 mM imidazole, pH 7.4 and dialyzed into PBS with 10% glycerol for storage while hGH was eluted in 50 mM Tris, 500 mM NaCl, 300 mM imidazole, 10% glycerol, pH 7.5 with 1 mM DTT, TCEP, and methionine to protect against oxidation and frozen directly for storage at -20 ºC. Protein concentrations were determined using the calculated absorbances at 280 nm.

When required, proteins were treated with additional FGE *in vitro* to fully convert Cys-to-fGly if intracellular conversion was sub-optimal. Briefly, aldehyde-tagged proteins (0.5 mg/mL) were incubated in 50 mM Tris, 100 mM NaCl, 0.1-1 mM β-Me, pH 9 with 0.05 equiv. *M. tuberculosis* FGE[6] for 20 h at rt. Proteins were then purified by size exclusion or buffer exchanged by spin concentration and stored in PBS.

Human serum albumin (HSA) and full-length human IgG aldehyde-tagged proteins were generated similar to previously described procedures.[7] Vectors bearing the heavy and light chain were constructed and nucleotides encoding the aldehyde tag were inserted at the C-terminus using standard molecular biology techniques. Protein production was performed by Redwood Bioscience using CHO-S cells overexpressing human FGE following standard protein expression protocols. HSA was purified from clarified media by Ni-NTA-agarose beads and eluted with high imidazole following typical His_6-tag purification procedures. Full-length human IgG was purified from clarified media by affinity chromatography using Protein-A agarose resin. The eluent was dialyzed into PBS and stored at -20 ºC. Cys-to-fGly conversion was assessed by standard addition on LCMS/MS of trypsinized IgG and demonstrated high conversion rates (Table S3).

Table S3. fGly conversion calculated on C-terminally aldehyde-tagged hIgG after *in vitro* conversion with additional FGE and analysis by LCMS/MS after trypsinization. Conversion rate was quantitated by standard addition of known amounts of aldehyde tag peptide fragments.

	Replicate* 1	Replicate 2	Replicate 3
Conversion Rate (%)	99.56%	99.60%	99.59%
Average Conversion Rate (%)		99.58%	
C.V.		0.02%	

c. Oxime formation and optimization

Alexa Fluor 488 C5-aminooxy-acetamide (AO-AF488) was purchased from Invitrogen and kept as a 10 mM stock. AO-FLAG [H_2NO-CH_2-CO-NH-(DYKDDDDK)-CO_2H] was custom synthesized by New England Peptide using standard Fmoc solid-phase peptide synthesis and stored as a 10 mM stock solution. Proteins were concentrated and buffer exchanged by dilution in Amicon Ultra-0.5 centrifugal filter devices (MWCO 10K or 30K, Millipore). Proteins were resolved by SDS-PAGE on Bis-Tris Criterion Gels (10% or 4-12%; Bio-Rad). Western blot analysis was performed by wet transfer (Tris-glycine, 20% MeOH) onto nitrocellulose at 100V for 60 min. Protein loading was confirmed by Ponceau stain (0.2% ponceau in 3% AcOH). Blocking and antibody incubation conditions were conducted in 1x Dulbecco’s phosphate buffered saline with 0.1% Tween-20 (PBST) with component concentrations as described.

Buffer pH: Aldehyde-tagged MBP was reacted at 25 µM with 400 µM AO-AF488 in 20 µL of buffer at 37 ºC for 18 h. Aldehyde-tagged hIgG was reacted at 20 µM with 500 µM AO-AF488 also in 20 µL of buffer at 37 ºC for 18 h. Buffer conditions are listed in Table S1 and made to the indicated concentration by dilution from a 10x stock in ddH_2O. Upon completion, the reactions were quenched with the addition of 4 µL 1M Tris, pH 7.5 and a 4 µL aliquot was taken for SDS-PAGE analysis. The remaining labeled protein was diluted into PBS, pH 7.4 and washed 4 times by concentration to remove unconjugated dye. The degree of labeling (DOL, % conjugate) was calculated by ratio of dye/protein concentration determined by UV-Vis absorbance (NanoDrop; AF488 at 494 nm; protein at 280 nm). An 11% correction was subtracted from the A_{280} based on the A_{494} reading to account for the absorbance overlap of Alexa Fluor 488 at 280 nm. Gels were scanned for fluorescence by a Typhoon 9410 imaging system (Amersham) before Coomassie stain to access protein loading.

Time and temperature: 5 µM of aldehyde-tagged hIgG was treated with 200 µM AO-CF488A (Biotium) in 40 µL of 100 mM KOAc, pH 4.6 and incubated at rt or 37 ºC. At 14, 24, 40, and 62 h, a 5 µL aliquot was taken and quenched by the addition of 1 µL 1 M Tris, pH 7.5. Aliquots were kept at -20 ºC until analysis at which 4x loading dye was added and loaded onto SDS-PAGE. Relative fluorescence was measured using ImageJ imaging software and given as fluorescence band density ratios after normalizing to coomassie stain density to control for loading.

Reagent concentration: 10 µM aldehyde-tagged hIgG was incubated at rt for 16 h in 10 µL of 5% MeCN, 0.02% FA with varying concentrations of AO-FLAG. Upon completion, the

reactions were probed by western blot (5% milk/PBST; anti-FLAG M2, Sigma, 1/1000; anti-mIgG-AP, Jackson Immunolabs, 1/1000) and visualized by Western Blue (Promega).

d. Linker and oxime vs. Cu-free click conjugations

DIBAC-488 was purchased from Click Chemistry Tools and kept as a 10 mM stock in DMSO. Linkers **1-3** and DIBAC-FLAG were generated as described and kept at 10 mM in ddH_2O. Protein loading was confirmed by Coomassie or Ponceau stain (0.2% ponceau in 3% AcOH). Blocking and antibody incubation conditions were conducted in 1x Dulbecco's phosphate buffered saline with 0.1% Tween-20 (PBST) with component concentrations as described. Membranes were developed by chemiluminescence using the SuperSignal West Pico kit (Thermo) or scanned for fluorescence by a Typhoon 9410 imaging system (Amersham). MALDI-TOF MS (Matrix-assisted laser desorption/ionization (time-of-flight) mass spectrometry) analysis was carried out on an Applied Biosystems Voyager DE-Pro machine. Typically, samples were diluted 8x in a saturated Sinapinic/Sinapic acid solution of 60% MeCN, 0.2% FA and 1 µL was deposited and dried under vacuum on a stainless steel sample plate (Applied Biosystems).

Fluorophore conjugation: Aldehyde-tagged hIgG at 5 µM was reacted with 0, 150, or 250 µM aminooxy compound (AO-AF488 or linkers **1-3)** in 10 µL 5% MeCN, 0.02% FA for 16 h at rt. To the linker conjugations was added 250 or 400 µM DIBAC-488 and incubated for an additional hour at 37 ºC, though reactions were later found to occur equally well at rt. To the reactions was then added 2 µL of 1 M Tris, pH 7.5, 4x loading dye, and samples were run on a 4-12% SDS-PAGE gel.

To compare yields among linkers at varying concentrations, 30 µM of aldehyde-tagged MBP or hGH was incubated with 300 or 750 µM of linker **1**, **2**, or **3** in NaH_2PO_4/NaCit buffer, pH 4.5 at 32 ºC for 18 h. Reactions were then supplemented with 500 µM or 1 mM DIBAC-488 and incubated at 4 ºC for 18 h before SDS-PAGE. Alternatively, the 750 µM linker reactions were diluted into PBS and concentrated 3 times to remove excess aminooxy linker. 15 µM of linker conjugated azide MBP or hGH was further reacted in PBS with 30 µM DIBAC-488 at 4 ºC for 18 h. Before SDS-PAGE, aliquots were taken for analysis by MALDI-TOF MS. Percent conjugation was determined by comparing peak heights of unreacted aldehyde-tagged protein to fluorophore conjugated peaks (+634 Da for DIBAC-488).

FLAG peptide conjugations: 50 µM MBP was reacted with 1 mM aminooxy linkers **1, 2,** or **3** in 20 µL of 100 mM KOAc, pH 4.6 for 16 h at 37 ºC. The reactions were then diluted into PBS and concentrated 3 times to remove excess aminooxy linker. 10 µM aldehyde-tagged MBP was reacted with 100 or 500 µM AO-FLAG or DIBAC-FLAG in 100 mM KOAc, pH 4.6 at 37 ºC. The linker labeled MBP was also reacted at 10 µM with 100 or 500 µM DIBAC-FLAG in PBS, pH 7.4 at rt. Reactions were flash frozen at 2 h or run for 16 h before analysis by western blot. Transferred nitrocellulose was probed with anti-FLAG M2-HRP (Sigma, 1/20000) in 5% milk/PBST and developed by film exposure with chemiluminescence.

To compare FLAG conjugations by oxime versus Cu-free click reaction over time, MBP and hGH were first preconjugated with linker **1** as previously described. 20 µM aldehyde-tagged protein was incubated with 40 or 400 µM AO-FLAG in 100 mM MES, pH 4.6 at 37 ºC or azide linker labeled protein was incubated with 40 or 400 µM DIBAC-FLAG in 10 µL PBS at rt. Aliquots were taken at the specified times, diluted with sinapinic acid matrix solution and dried on the MALDI plate for mass spec analysis. Percent conjugation was determined by comparing

peak heights of unreacted aldehyde-tagged protein to FLAG conjugated peaks (+1068 Da for AO-FLAG; +1580 Da for DIBAC-FLAG).

e. Protein-protein conjugations, flow cytometry and TEM

Aldehyde-tagged proteins at 25-75 µM were first reacted with 800 µM aminooxy linkers **1** or **4** in NaCit/NaH_2PO_4 buffer, 1 mM EDTA, 2% DMSO, pH 4.5 for 14 h at 35 ºC. Reactions were quenched by dilution into PBS, pH 7.4 and concentrated 3 times by filtration to remove excess linker. Conjugation was confirmed by MALDI-TOF MS to ensure greater than 75% oxime yield. Cu-free click reactions were performed with 2 equiv of one partner in PBS at 4 ºC for 16 h. Shorter reaction times at rt were performed though were lower yielding. Reactions with DIBAC-488 or Az-647 (Invitrogen) were imaged by fluorescence scan on a Typhoon.

In large scale conjugations, 80 µM azide linker **1** labeled hGH or MBP was incubated with 20 µM DIBAC linker **4** labeled hIgG (40 µM available aldehyde, one per C-term) in 25 µL of PBS at 4 ºC for 20 h. 2 µL were retained for further analysis and the remaining crude mixture was purified by hydrophobic interaction chromatography (HIC) followed by size exclusion chromatography (SEC). HIC was performed on a ProPac HIC-10 column (5 µm, 100 x 4.6 mm, Dionex) and eluted in a linear gradient of 800 mM Na_2SO_4, 25 mM NaH_2SO_4, pH 5.8 to 25 mM NaH_2SO_4, 10% MeCN, pH 5.8 at 0.8 mL/min. Fractions were collected based on absorbtion at 215 nm and 280 nm, concentrated and buffer exchanged into 25 mM Tris, 100 mM NaCl, pH 7.5. Protein concentration of the collected fractions were calculated based on the A_{280} and further analyzed by western blot to confirm presence of protein chemical conjugates. To further clean the protein samples for TEM imaging, SEC was performed on a Superdex 200 10/300 GL column (GE Healthcare) and eluted in an isocratic run of 25 mM Tris, 100 mM NaCl, pH 7.5 at 0.5 mL/min. Elutions were monitored at A_{215}, collected fractions were concentrated, and protein concentration was determined by A_{280}.

The click protein chemical conjugates were analyzed by western blot against unconjugated starting protein, unpurified crude reaction, and a negative control. The negative control consisted of pre-reacted linkers **1** and **4** which were incubated at 1 mM with 80 µM hGH or MBP and 20 µM hIgG in NaCit/NaH_2PO_4 buffer pH 4.5 for 16 h at 35 ºC. This demonstrated the inability of oxime formation to generate protein oligomers. The samples were run on 4-12% SDS-PAGE and blotted onto nitrocellulose at 100 V for 2 h at 4 ºC. Ponceau staining was performed to confirm protein loading and densitometry measurements were taken using the program ImageJ to demonstrate conjugation yields of over 70%. Presence of MBP or hGH in the higher MW conjugates was confirmed by probing with mouse anti-hGH (Abcam, 1/5000) or mouse anti-MBP (New England Biolabs, 1/30000) in 3% milk/PBST followed by anti-mIgG-FITC (Jackson Immunolabs, 1/4000) in 5% BSA/PBST and scanned for fluorescence. hIgG was visualized by probing with goat anti-hIgG-DyLight 649 (Jackson Immunolabs, 1/4000) in 5% BSA/PBST and scanned for fluorescence.

Cell binding and flow cytometry: SKOV3 ovarian cancer cells and Jurkat T cells were purchased from ATCC and cultured in RPMI-1640 media supplemented with 10% fetal calf serum (FCS) and penicillin/streptomycin. Upon reaching confluency, cells were lifted with EDTA/trypsin (SKOV3), harvested (Jurkats), centrifuged at 300xg, and resuspended in FACS buffer (1% FCS in PBS) at $1x10^6$ cells/mL. Protein chemical conjugates were generated by incubating 1 equivalent of each protein click partner (5 µM α-HER2hIgG-DIBAC/hGH-Az, α-HER2hIgG-DIBAC/MBP-Az, or each alone) in PBS at 4 ºC for 18 h. Aliquots of 150 µL cells

($1x10^6$ cells/mL in FACS) were incubated with a 1/500 dilution of the click reactions in triplicate on ice for 45 min. Cells were centrifuged at 350xg and washed 3 times in FACS buffer. Cells were then incubated on ice for 30 min with mouse anti-hGH (Abcam, 1/500) or mouse anti-MBP (New England Biolabs, 1/500) followed by 30 min incubations with goat anti-hIgG-DyLight 649 (Jackson Immunolabs, 1/2000) to detect α-HER2 hIgG binding or goat anti-mIgG-FITC (Jackson Immunolabs, 1/500) to detect hGH/MBP cell surface delivery. Samples were resuspended in 300 µL of FACS buffer and analyzed on a BD Biosciences FACSCalibur flow cytometer with analyses performed using FlowJo software (Tree Star).

TEM and data processing: Protein chemical conjugations (MBP-hIgG) were diluted to a final concentration of 26 nM in 20 mM Tris pH 7.4, 100 mM NaCl buffer. 4 ul of sample were deposited on a glow-discharged, carbon coated 400 mesh copper grid (EM Sciences). After 1 min incubation, the sample was stained in 4 successive 75 µl drops of 1% uranyl formate stain on Parafilm and blotted dry. Data was collected in a Tecnai 12 TEM (FEI) operated at 120 kV at 49,000x nominal magnification on a TemCam F416 camera (TVIPS), with a pixel size corresponding to 0.22 nm at the specimen scale. 4,200 MBP-hIgG particles were selected semi-automatically using boxer (EMAN package).[8] Single particle data analysis was done using an automatic 2D alignment and classification scheme, combining cycles of an in-house implementation of the self-organizing network classification (V.H. Ramey, http://cryoem.berkeley.edu)[9] and of a classical multi-reference alignment (MRA) in IMAGIC (Image Science Software GmbH, Berlin, Germany).[10] The final classification grouped the data aligned in 2D into 24 classes. Class average images were compared to published IgG and MBP structures to identify densities. The crystal structures of a mouse IgG (PDB 1IGY) and MBP (1JW4) were manually docked onto the EM 2D density maps at the same scale in Chimera[11] (supported by NIH grant P41 RR-01081).

III. Supporting References

[1] I. S. Carrico, B. L. Carlson, C. R. Bertozzi, *Nat. Chem. Biol.* **2007**, *3*, 321-322.

[2] K. D. Park, R. Liu, H. Kohn, *Chem. Biol.***2009**, *16*, 763-772.

[3] A. M. Bondia, N. Larcher, L. Garrelly, J. C. Rossi, R. Pascal, *Tetrahed. Lett.* **2010**, *51*, 3330-3333.

[4] M. Rashidian, J. K. Dozier, S. Lenevich, M. D. Distefano, *Chem. Commun.* **2010**, *46*, 8998-9000.

[5] D. S. Jones, K. A. Cockerill, C. A. Gamino, J. R. Hammaker, M. S. Hayag, G. M. Iverson, M. D. Linnik, P. A. McNeeley, M. E. Tedder, H.-T. Ton-Nu, et al., *Bioconjugate Chem.* **2001**, *12*, 1012-1020.

[6] B. L. Carlson, E. R. Ballister, E. Skordalakes, D. S. King, M. A. Breidenbach, S. A. Gilmore, J. M. Berger, C. R. Bertozzi, *J. Biol. Chem.* **2008**, *283*, 20117-20125.

[7] P. Wu, W. Shui, B. L. Carlson, N. Hu, D. Rabuka, J. Lee, C. R. Bertozzi, *Proc. Natl. Acad. Sci. USA* **2009**, *106*, 3000-3005.

[8] S. J. Ludtke, P. R. Baldwin, W. Chiu, *J. Struct. Biol.***1999**, *128*, 82-97.

[9] T. Ogura, K. Iwasaki, C. Sato, *J. Struct. Biol.* **2003**, *143*, 185-200.

[10] M. van Heel, G. Harauz, E. V. Orlova, R. Schmidt, M. Schatz, *J. Struct. Biol.* **1996**, *116*, 17-24.

[11] E. F. Pettersen, T. D. Goddard, C. C. Huang, G. S. Couch, D. M. Greenblatt, E. C. Meng, T. E. Ferrin, *J. Comput. Chem.* **2004**, *25*, 1605-1612.
